# 248. Change in Incidence of Multisystem Inflammatory Syndrome in Children Across the COVID-19 Pandemic in Chicago — March 2020 – March 2022

**DOI:** 10.1093/ofid/ofac492.326

**Published:** 2022-12-15

**Authors:** Hillary Spencer, Jennifer Seo, Massimo Pacilli, Candice L Robinson, Hannah Matzke, Maria Joseph, Stephanie Gretsch

**Affiliations:** CDC, Chicago Department of Public Health, Chicago, IL; Chicago Department of Public Health, Chicago, Illinois; Chicago Department of Public Health, Chicago, Illinois; Chicago Department of Public Health, Chicago, Illinois; Chicago Department of Public Health, Chicago, Illinois; Chicago Department of Public Health, Chicago, Illinois; Chicago Department of Public Health, Chicago, Illinois

## Abstract

**Background:**

Peak counts of multisystem inflammatory syndrome in children (MIS-C) have followed each COVID-19 peak by 2–5 weeks. Fewer cases of MIS-C occurred after the Delta-predominant period compared to early waves of the pandemic. The Chicago Department of Public Health analyzed the ratio of MIS-C to pediatric COVID-19 hospitalization by period of variant predominance from March 2020 – mid-March 2022 to evaluate differences by variant.

**Methods:**

MIS-C in Chicago residents was reported using the standard CDC MIS-C case report form. Four periods of COVID-19 infection were identified with dates defined by variant predominance (≥50%); date ranges for corresponding MIS-C periods were defined as starting 21 days after the COVID-19 period (Table 1). MIS-C cases were compared to hospitalizations as a measure of COVID-19 disease activity in children (rather than case counts which are more subject biases inherent in disease testing) among confirmed and probable COVID-19 cases in children ≤21 years reported to CDPH. Ratios of MIS-C cases per 100 corresponding pediatric hospitalizations for each variant period were calculated. Proportions of MIS-C hospitalizations with intensive care unit (ICU) admission, mechanical ventilation (MV), and receipt of vasopressors (VP) were calculated as markers of disease severity in MIS-C for each wave.

**Figure:**
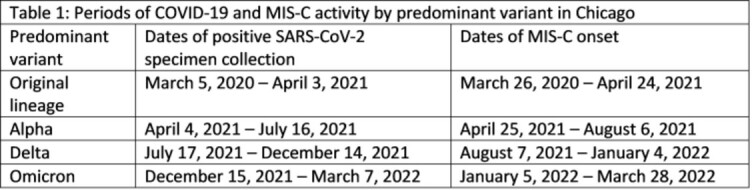

**Results:**

90 cases of MIS-C and 1,597 pediatric COVID-19 hospitalizations were reported (Table 2). The overall ratio was 5.6 MIS-C cases/100 pediatric COVID-19 hospitalizations. The first period with predominance of the original lineage had a ratio of 10.2; subsequent periods with variant predominance all had lower ratios. The Delta period had the second highest ratio which was 68% of the first period. Across waves, 74% of MIS-C patients were admitted to ICU (range, 67–82%); 11% underwent MV (range, 0–14%); and 52% received VP (range, 45–71%).

**Figure:**
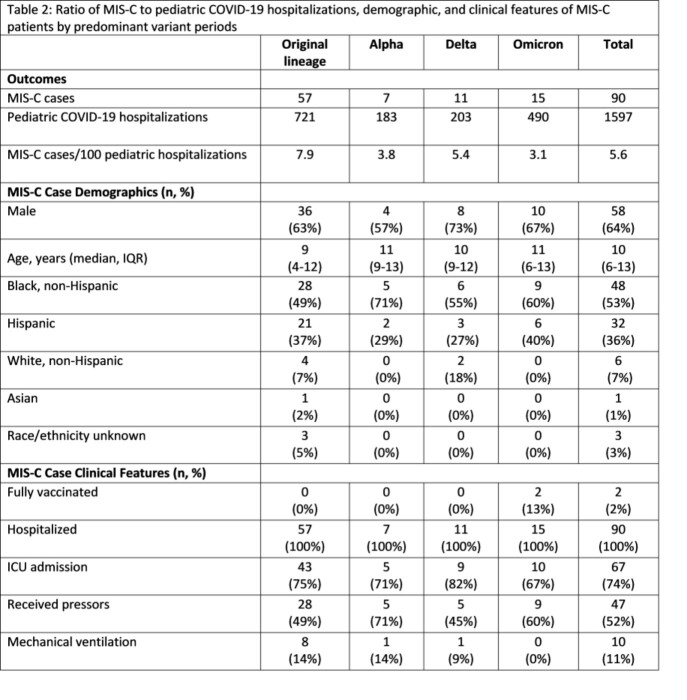

**Conclusion:**

The ratio of MIS-C to pediatric COVID-19 hospitalizations varied by period of SARS-CoV-2 variant predominance. The ratio was highest in the early pandemic. There was no consistent change in MIS-C severity. Further study is needed to determine if the change in ratio is due to increased immunologic exposure (vaccination or previous infection) or if it is variant dependent.

**Disclosures:**

**All Authors**: No reported disclosures.

